# Comparative transcriptome profiling reveals differential defense responses among *Alternaria brassicicola* resistant *Sinapis alba* and susceptible *Brassica rapa*

**DOI:** 10.3389/fpls.2023.1251349

**Published:** 2024-01-18

**Authors:** Reshma Ahmed, Kuntal Kumar Dey, Muthappa Senthil-Kumar, Mahendra Kumar Modi, Bidyut Kumar Sarmah, Priyadarshini Bhorali

**Affiliations:** ^1^ Department of Agricultural Biotechnology, Assam Agricultural University, Jorhat, Assam, India; ^2^ National Institute of Plant Genome Research, New Delhi, India; ^3^ Department of Biotechnology - Northeast Centre for Agricultural Biotechnology, Assam Agricultural University, Jorhat, Assam, India

**Keywords:** Alternaria blight, *Alternaria brassicicola*, *Sinapis alba*, transcriptome profiling, resistance, defense

## Abstract

Alternaria blight is a devastating disease that causes significant crop losses in oilseed Brassicas every year. Adoption of conventional breeding to generate disease-resistant varieties has so far been unsuccessful due to the lack of suitable resistant source germplasms of cultivated *Brassica* spp. A thorough understanding of the molecular basis of resistance, as well as the identification of defense-related genes involved in resistance responses in closely related wild germplasms, would substantially aid in disease management. In the current study, a comparative transcriptome profiling was performed using Illumina based RNA-seq to detect differentially expressed genes (DEGs) specifically modulated in response to *Alternaria brassicicola* infection in resistant *Sinapis alba*, a close relative of Brassicas, and the highly susceptible *Brassica rapa*. The analysis revealed that, at 48 hpi (hours post inoculation), 3396 genes were upregulated and 23239 were downregulated, whereas at 72 hpi, 4023 genes were upregulated and 21116 were downregulated. Furthermore, a large number of defense response genes were detected to be specifically regulated as a result of Alternaria infection. The transcriptome data was validated using qPCR-based expression profiling for selected defense-related DEGs, that revealed significantly higher fold change in gene expression in *S. alba* when compared to *B. rapa*. Expression of most of the selected genes was elevated across all the time points under study with significantly higher expression towards the later time point of 72 hpi in the resistant germplasm. *S. alba* activates a stronger defense response reaction against the disease by deploying an array of genes and transcription factors involved in a wide range of biological processes such as pathogen recognition, signal transduction, cell wall modification, antioxidation, transcription regulation, etc. Overall, the study provides new insights on resistance of *S. alba* against *A. brassicicola*, which will aid in devising strategies for breeding resistant varieties of oilseed Brassica.

## 1 Introduction

Alternaria blight, also known as Alternaria leaf spot is one of the most destructive diseases of oilseed Brassicas all over the world. The disease results in yield losses of up to 50% and is becoming a major threat to Brassica species (Jyoti et al., 2021). In India, it has been reported to cause losses of up to 70% (Kumar et al., 2016). Alternaria blight is mainly caused by two necrotrophic fungi, *Alternaria brassicicola* and *A. brassicae*, that are mostly found co-inhabiting the same plant. These pathogens are influenced by climatic conditions with their highest occurrence during the winter season (Humpherson-Jones and Phelps, 1989). The disease affects the aerial parts of the plant at all stages of growth including the siliquae and seeds and, at advanced stages of infection, leads to complete decay of the whole plant (Meena et al., 2016; Mandal et al., 2018; Macioszek et al., 2020). Infection by *A. brassicicola* initially results in the appearance of tiny blackish spots on the lower leaves, that later enlarge to develop distinct, round spots of varying sizes with yellow halos and concentric rings (Meena et al., 2016). The disease reduces the photosynthetic potential of plants, affecting normal growth thereby resulting in lowered oil content (Saharan et al., 2016). Thus, for reviving the yield potential of oilseed Brassicas, management of the disease is one of the foremost concerns. Management of Alternaria blight mostly relies on the application of fungicides, but the use of broad-spectrum chemicals poses serious threat to the environment besides resulting in the development of resistance in pathogens. Adoption of conventional breeding strategies to develop resistant cultivars against the disease is confounded due to non-availability of suitable resistance sources within the available germplasm of cultivated species of Brassica. Among the oilseed Brassicas, *Brassica rapa* is the most susceptible to Alternaria blight (Aneja and Agnihotri, 2016). However, *Sinapis alba*, a closely related wild species belonging to the Brassicaceae family, is reported to show resistance against the Alternaria pathogens (Hansen and Earle, 1997; Mazumder et al., 2013; Fatima et al., 2019a; Yadav et al., 2020). Reports state that close genetic relationship and the ease of forming hybrids between *S. alba* and *Brassica* make it a potential donor of resistance and other agronomic traits to Brassica crops (Brown et al., 1997; Jiang et al., 2013a).

An interaction between a plant and a pathogen can be compatible or incompatible involving a series of events, starting from recognition of the pathogen to development of a response in the plant. Pathogens secrete specific effectors molecules in order to establish pathogenicity in response to which, the host plant derived metabolic products trigger a defense response. Generally, a plant system activates two forms of defenses in response to pathogen attack. Initially, the basal response is activated that involves recognition of pathogen specific effector molecules such as fungal chitin, bacterial flagellins, lipopolysaccharides etc., together known as pattern recognition receptors (PRRs), which leads to activation of PAMP (pathogen associated molecular pattern)- triggered immunity (PTI). Subsequently, the plant activates a second line of defense which involves the host specific resistance (R) genes that can identify effectors and, can be perceived by the pathogen avirulence (Avr) genes, resulting in effector- triggered immunity (ETI) (Flor, 1971; Albersheim and Anderson-Prouty, 1975; Jones and Takemoto, 2004). PTI and ETI result in a range of downstream responses such as reactive oxygen species (ROS) burst, calcium influx, phytohormone mediated signal transduction, closure of stomata, modification of cell wall and biosynthesis of a varied set of secondary metabolites and antimicrobials, that prevent or restrict pathogen spread (Meng and Zhang, 2013; Andersen et al., 2018; Peng et al., 2018).

Several studies are being carried out worldwide to explore plant-pathogen interactions and understand mechanisms of pathogenicity and host defense in different plant species including oilseed Brassicas. The development of new technologies to carry out genome-wide studies, the accessibility of genomic and sequence data and the development of data analysis platforms have greatly facilitated the characterization of transcriptomic responses associated with plant-pathogen interactions. Transcriptome analysis based on RNA sequencing (RNA-seq) has led to a considerable understanding of molecular mechanisms underlying specific biological processes that result in pathogenesis or host resistance (Meng et al., 2021a; Borah et al., 2022). Studies on *B. rapa* infected with *Plasmodiophora brassicae* have detected differential expression of genes associated with effector recognition, Ca^2+^ influx, cell wall modification, transcription factors, pathogenesis related (PR) genes, etc. (Chu et al., 2014; Chen et al., 2016). Similarly, transcriptomics has been utilized to identify genes associated with resistance to *Leptosphaeria maculans* infection in *B. napus* (Becker et al., 2017; Becker et al., 2019). A global RNA-seq study was carried out to identify gene functions at the time of initial infection by *Sclerotinia sclerotiorum* on susceptible *B. napus* cv. Westar and resistant *B. napus* cv. Zhongyou 821 plants (Girard et al., 2017), which detected ethylene responsive factors associated with host resistance. Lately, Chittem et al. (2020) performed transcriptome profiling in order to understand the molecular basis of *B. napus* and *S. sclerotiorum* interaction. They carried out differential gene expression analysis during the establishment of stem rot disease on two canola lines varying in susceptibility to the pathogen. Recently, transcriptome expression profiling was done in *B. juncea* during growth and infection by *A. brassicae* which revealed that 4,430 genes were differentially expressed during infection (Rajarammohan, 2023).

Development of disease resistant varieties is one of the major challenges of sustainable crop production. Exploring and utilizing novel sources of resistance such as the wild or close relatives, and identification of defense related genes involved in resistance response from such species, would greatly contribute towards management of phytopathogens. The transfer of genes from germplasms such as *S. alba* to cultivated *Brassica* spp. is a highly potential approach towards development of varieties resistant to *Alternaria* blight. In order to achieve this, it is not only essential to dissect the mechanisms underlying the defense pathways, but identification and characterization of resistance-related candidate genes is a pre-requisite. With this background, in a previous study, we attempted to screen host resistance in *S. alba* against the necrotrophic pathogen *A. brassicicola* and characterize the *S. alba - A. brassicicola* interaction through pathogenicity and morpho-histopathological studies using *B. rapa- A. brassicicola* interaction as a reference. The study had revealed *S. alba* to be considerably resistant to *A. brassicicola* whereas *B. rapa* as expected, was extremely susceptible. We therefore hypothesized that, a comparative transcriptomic analysis among these resistant and susceptible germplasms would be able to identify specific genes involved in defense against the Alternaria pathogen. In this study, we performed a comprehensive RNA-seq based analysis to elucidate the *A. brassicicola* induced defense responses in *S. alba*, in comparison with the highly susceptible cultivar *B. rapa* var. Toria across different time points after pathogen inoculation. The investigation revealed that the differences between resistance and susceptibility were associated with the magnitude of expression changes in a suite of genes involved in pathogen recognition, signaling cascades, cell wall modification, antioxidation, transcription regulation, biosynthesis of defense-related proteins, etc. The results were supported by quantification of expression levels of some potential candidate defense-related genes. Overall, the research provides insights on the intricate molecular processes behind *S. alba's* immune response against *A. brassicicola* and offers a useful resource for devising efficient strategies of disease management.

## 2 Materials and methods

### 2.1 Plant materials and artificial pathogen inoculation

For the present investigation, two sets of plant materials were used: *B. rapa* var. Toria (variety TS-38) which is highly susceptible and, *S. alba* which is resistant to Alternaria blight. The seeds of *B. rapa* and *S. alba* were procured from the Regional Agricultural Research Station, Nagaon, Assam, and the National Bureau of Plant Genetic Resources, New Delhi, respectively. The seeds were treated with 70% ethyl alcohol for 2 min, rinsed with sterile double distilled water, and then surface sterilized with 4% sodium hypochlorite for 10 min and, finally rinsed three times with sterile double distilled water. After soaking on a sterile filter paper, the seeds were sown in small plastic pots containing a mixture of cocopeat and vermiculite (3:1) under a greenhouse. The experiment was done in 3 replicates with each pot containing 3 plants. At 5-6 leaf stage, the plants were transferred to individual pots and maintained in a growth chamber at a temperature of 23^°^C, 90% relative humidity and light intensity of 12.5 µmol m^-2^ s^-1^ with 16 h/8 h light/dark cycle for 7 days until artificial inoculation.

The pure cultures of *A. brassicicola* isolated from diseased leaf tissues of *B. rapa* were used to artificially inoculate both the sets of susceptible and resistant plants. For inoculation, *A. brassicicola* spore suspension was prepared using well sporulating cultures growing on potato dextrose agar (PDA) medium in petri-plates, using sterile double distilled water and the concentration was adjusted to 5×10^4^ spores ml^-1^ (Doullah et al., 2006). The spore suspension was sprayed on both sides of the leaves and then the complete plant, and the plants used as control were sprayed with sterile double distilled water. The plants were covered with polybags for 24 hours to maintain humidity. Leaf tissues were collected from the plants at different time points namely, 0, 24, 48 and, 72 hours post-inoculation (hpi) from both, pathogen inoculated (treated) and mock-inoculated (control) sets of plants, in at least three biological replicates. The leaf samples collected were immediately immersed in liquid nitrogen and later stored at -80°C.

### 2.2 Isolation of total RNA, library preparation and sequencing

Total RNA was extracted from the leaf samples collected after artificial pathogen inoculation i.e., from both sets of tissues (resistant and susceptible) collected at 0, 24, 48 and, 72 hpi using TRIzol^®^ reagent (Invitrogen™, USA) following standard instructions. The RNA samples were treated with 1 μl of 2U DNase I (RNase-free) (Invitrogen™, USA) by incubating at 37°C for 30 min to eliminate DNA. To check the quality of the isolated total RNA, the samples were electrophoresed in a denaturing 1% agarose gel. The concentration and purity of the extracted RNA were checked using a NanoDrop™ One/OneC Microvolume UV-Vis spectrophotometer (Thermo Fisher Scientific, USA). At least three RNA samples were pooled from each set of experiment for all the time points. The total RNA, pooled from three biological replicates, extracted from the tissues at two time points namely, 48 and 72 hpi, were outsourced to Bencos Research Solutions Pvt. Ltd., Mumbai, for transcriptome sequencing using the Illumina NovaSeq 6000 Platform.

### 2.3 RNA-seq analysis and identification of differentially expressed genes

The raw reads obtained after sequencing were subjected to standard quality control and, the adaptors and low-quality sequences (Q<30) were trimmed using the Cutadapt tool (Martin, 2011). The quality reads generated were mapped to the Alternaria genome using HISAT2 to check and remove the reads of fungal origin. The unmapped reads were extracted and mapped with the *B. rapa* reference genome by using the HISAT2 splice aligner tool (Kim et al., 2019) and per sample read counts were generated using Cufflink. The read counts were then subjected to differential gene expression analysis using Cuffdiff. The grouping of the samples for DEG analysis was done based on the two selected time points, namely 48 hpi and 72 hpi, at which the gene expression of the susceptible and resistant sets of plants were detected. To differentiate between significantly upregulated and downregulated genes, log2 fold change ≥+1.5 and ≤-1.5 were considered. Heatmap was generated for data quality assessment by sample clustering and visualization of top 100 upregulated DEGs on the basis of their normalized read count information. Gene ontology (GO) enrichment analysis of DEGs was performed with g:Profiler (Reimand et al., 2011).

### 2.4 Quantitative PCR based validation and gene expression profiling

For qPCR studies, the first strand cDNAs were synthesized from 1 µg total RNA extracted from each of the collected tissue sample by using the PrimeScript™ RT kit with gDNA Eraser (Takara Bio USA, Inc.) following manufacturer’s instructions. Primers for selected genes were designed using the PrimerQuest Tool by IDT. Expression analysis of 8 selected DEGs was performed in the Real-Time PCR system Quant studio 5 (Applied Biosystems, USA), using TB Green Premix Ex Taq II (Tli RNase H Plus) (2X) (Takara Bio USA, Inc.). Each reaction mixture consisted of 5 μl 2X TB Green Premix Ex Taq II, 10 μM forward and reverse primers (gene specific), appropriate volume of cDNA (10 ng) and nuclease free water in a total volume of 10 μl. The PCR profile followed was: initial denaturation at 95°C/10 min, followed by 30 cycles of denaturation at 95°C/15 sec, annealing at gene specific temperature (60°C) for 30 sec and, finally extension at 72°C for 30 sec. All experiments were performed twice by using three technical replicates and three biological replicates. Actin 2 was taken as the reference gene for the study. The details of the selected genes and primers used in the qPCR analysis are given in **Supplementary Table 1**. The relative fold change in gene expression was calculated using the 2^-ΔΔCT^ method (Livak and Schmittgen, 2001) and transformed to log2 value. Statistical analysis was done by two-way ANOVA in excel. The significant difference of relative expression level between the two germplasms at different time points was determined at p-value *p* < 0.05.

## 3 Results

### 3.1 Disease development

**Figure 1:**
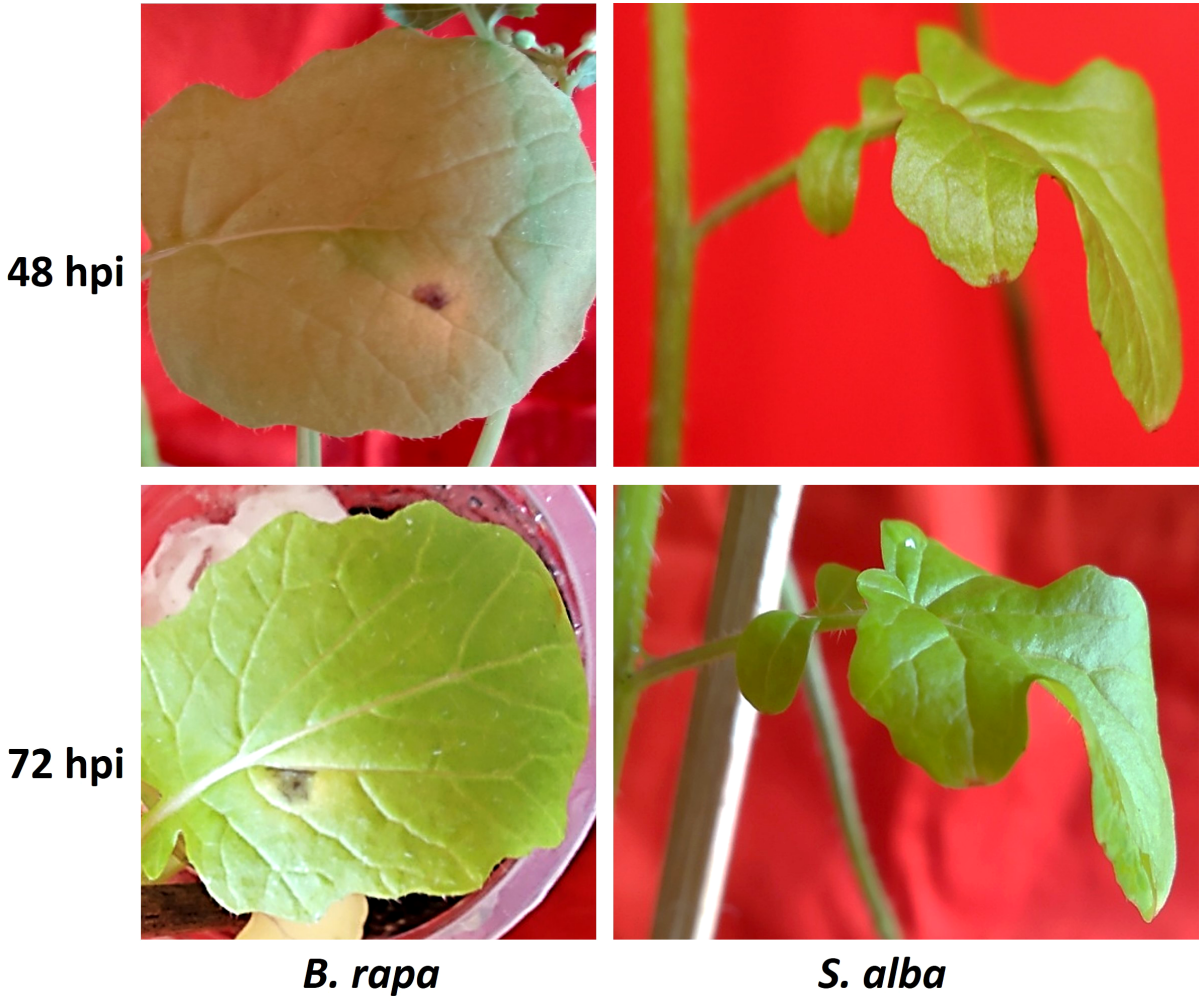
Symptom development on *B*. *rapa* (susceptible) and *S. alba* (resistant) inoculated with *A. brassicicola*. Distinct symptoms occurred at 48 hpi on *B*. *rapa* that became more prominent at 72 hpi but no visible symptoms appeared on resistant *S. alba* during these time points.

The differences in symptoms of Alternaria blight between the resistant *S. alba* and susceptible *B. rapa*, after artificial inoculation with *A. brassicicola*, were clearly apparent at all the time points under study (**Figure 1**). In *B. rapa*, the symptoms were seen as very small dark greyish brown to blackish spots on leaf surfaces at around 24 hpi. The symptoms became distinct within 48-72 hpi with a yellow halo surrounding the lesions. On the other hand, in case of *S. alba*, no symptoms were seen until 5-7 days post-inoculation (dpi). The necrotic spots on *B. rapa* grew over time and covered almost the entire leaf lamina within 7-10 dpi, whereas in *S. alba*, the spots did not grow any further even after 10 dpi.

### 3.2 RNA-seq, mapping and identification of differentially expressed genes

For RNA-seq experiments, paired end sequencing libraries were prepared from the pooled total RNA samples that passed the quality control test. The samples with RNA integrity number (RIN value) ≥ 8 as determined by Agilent Bioanalyzer 2100 were further chosen for library preparation. Binary data was converted into FASTQ utilizing Illumina package bcl2fastq (v.0.11.8). A total of 39,150,854 and 39,690,944 raw reads were obtained from *B. rapa* samples at 48 and 72 hpi respectively. From the *S. alba* samples, 37,040,439 and 42,696,304 raw reads were obtained at 48 and 72 hpi respectively. The numbers of quality reads for these samples were 38,644,791, 39,398,767, 36,528,092 and 42,004,916. Mapping of the reads with the pathogen genome revealed that around 0.8% of the reads mapped with Alternaria which were discarded, while the rest were used for analysis. The mapping percentages for *B. rapa* at 48 and 72 hpi were found to be 96.63% and 92.87% respectively, while those for *S. alba* at 48 and 72 hpi were 79.41% and 78.46%, respectively (**Supplementary Data Sheet 1**). The RNA-seq data has been submitted to the publicly-available repository of NCBI, Sequence Read Archive (SRA) (PRJNA784760).

**Figure 2:**
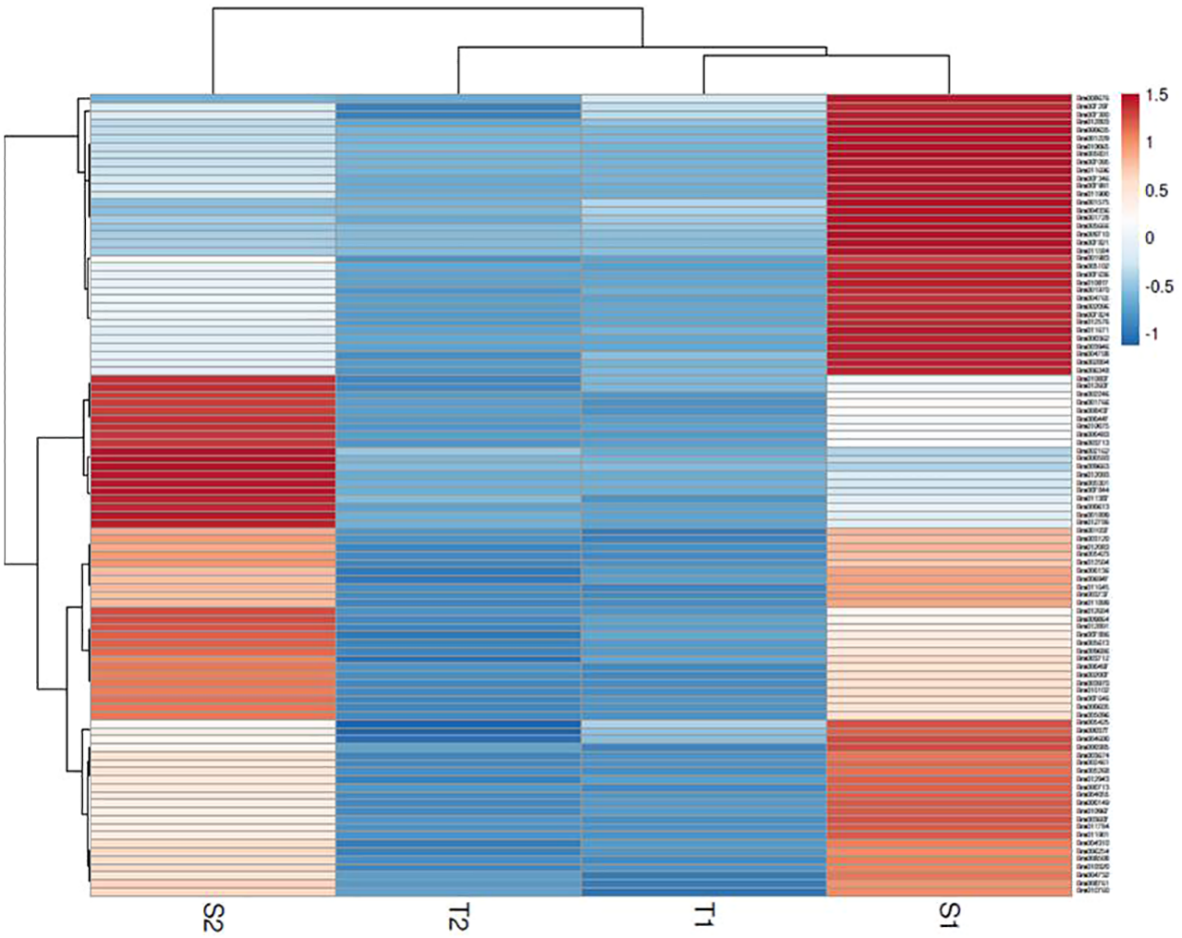
Heatmap of top 100 DEGs upregulated in response to *A*. *brassicicola* infection at 48 hpi and 72 hpi clearly depicting a distinct pattern of gene expression among the resistant and susceptible germplasms (S1- *S. alba* at 48 hpi; S2- *S. alba* at 72 hpi; T1- *B*. *rapa* at 48 hpi; T2- *B*. *rapa* at 72 hpi).

The assembly of the mapped reads with reference *B. rapa* genome revealed a vast set of genes at log2 fold change ±1.5, that resulted in the identification of 26635 DEGs at 48 hpi and 25139 DEGs at 72 hpi. A total of 3396 genes were found to be upregulated and 23239 genes were downregulated at 48 hpi while, 4023 and 21116 genes were upregulated and downregulated respectively at 72 hpi. The analysis clearly depicts that the number of genes downregulated was much higher than the number of upregulated genes. The enrichment data of genes differentially expressed during the host-pathogen interaction were further searched for common genes across the two time points. A total of 391 genes were upregulated and 5474 genes were downregulated commonly across 48 and 72 hpi (**Supplementary Data Sheet 2**). Out of 5865 genes that were found to be commonly expressed at 48 and 72 hpi, 52 genes were exclusively expressed in *S. alba* upon *A. brassicicola* infection. The heatmap analysis of top 100 upregulated genes revealed a high expression of genes in *S. alba* at 48 and 72 hpi in comparison to *B. rapa* in response to *A. brassicicola* infection (**Figure 2**).

### 3.3 Functional annotation and enrichment analysis

**Figure 3:**
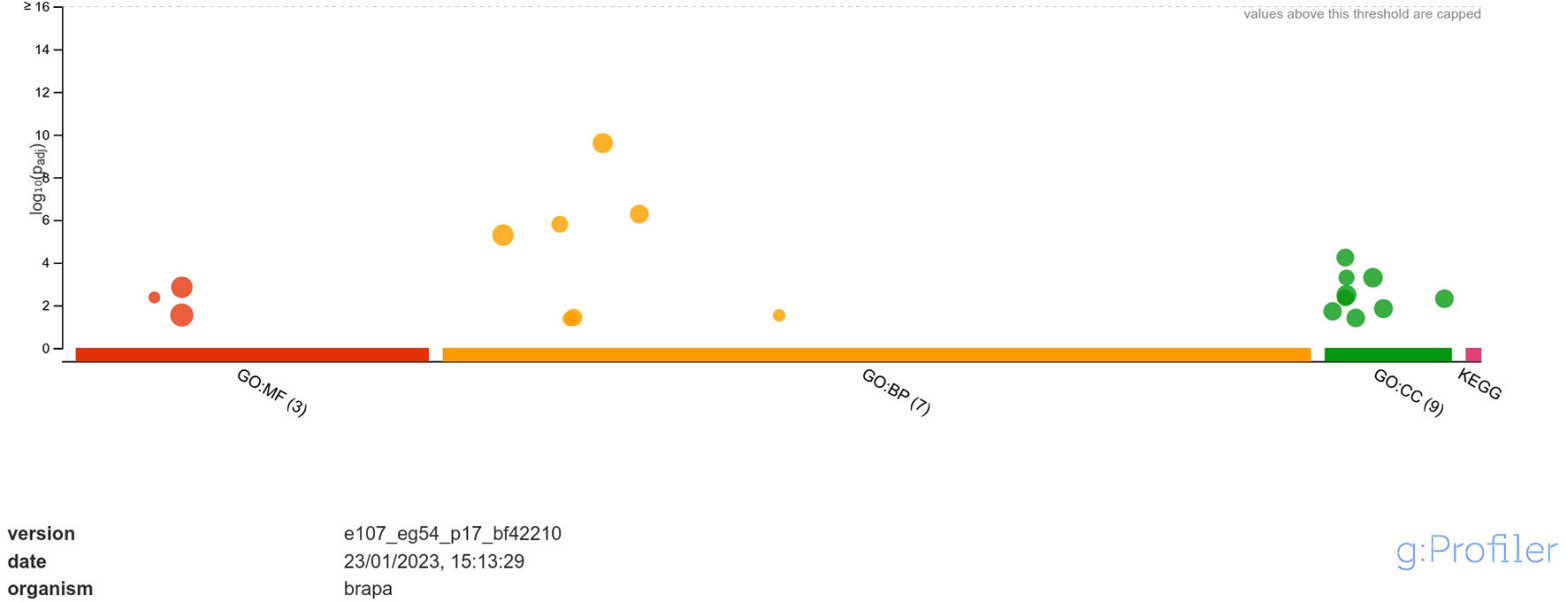
Functional enrichment analysis of DEGs upregulated in response to *A. brassicicola* infection, using g:Profiler. In the figure, the size of the circle represents the number of genes under the categories of MF, BP and CC.

Functional annotation through GO analysis annotated the DEGs regulated in response to *A. brassicicola* infection into different classes of molecular function (MF), biological process (BP) and, cellular component (CC) (**Supplementary Data Sheet 2**). The analysis revealed that the upregulated DEGs were annotated into 3 classes of MF namely, hexosyltransferase activity, oxygen evolving activity, glycosyltransferase activity; 7 classes of BP namely, photosynthesis, photosynthesis-light reaction, photosynthesis-light harvesting, generation of precursor metabolites and energy, photosystem II stabilization, xyloglucan metabolic process, photosystem II assembly and; 9 classes of CC namely, photosystem, photosystem II oxygen evolving complex, photosynthetic membrane, thylakoid, photosystem II, oxidoreductase complex, thylakoid membrane, cell wall and, external encapsulating structure (**Figure 3**). Among MF, the highly enriched classes were ‘glycosyltransferase activity’ and ‘hexosyltransferase activity’ with 20 and 15 annotated genes respectively. In case of BP, the most highly enriched classes were ‘photosynthesis’ and ‘generation of precursor metabolites and energy’ each with 20 annotated genes. Similarly, under CC, the most highly enriched classes were ‘photosynthetic membrane’ and ‘thylakoid’ each having 11 annotated genes.

**Figure 4:**
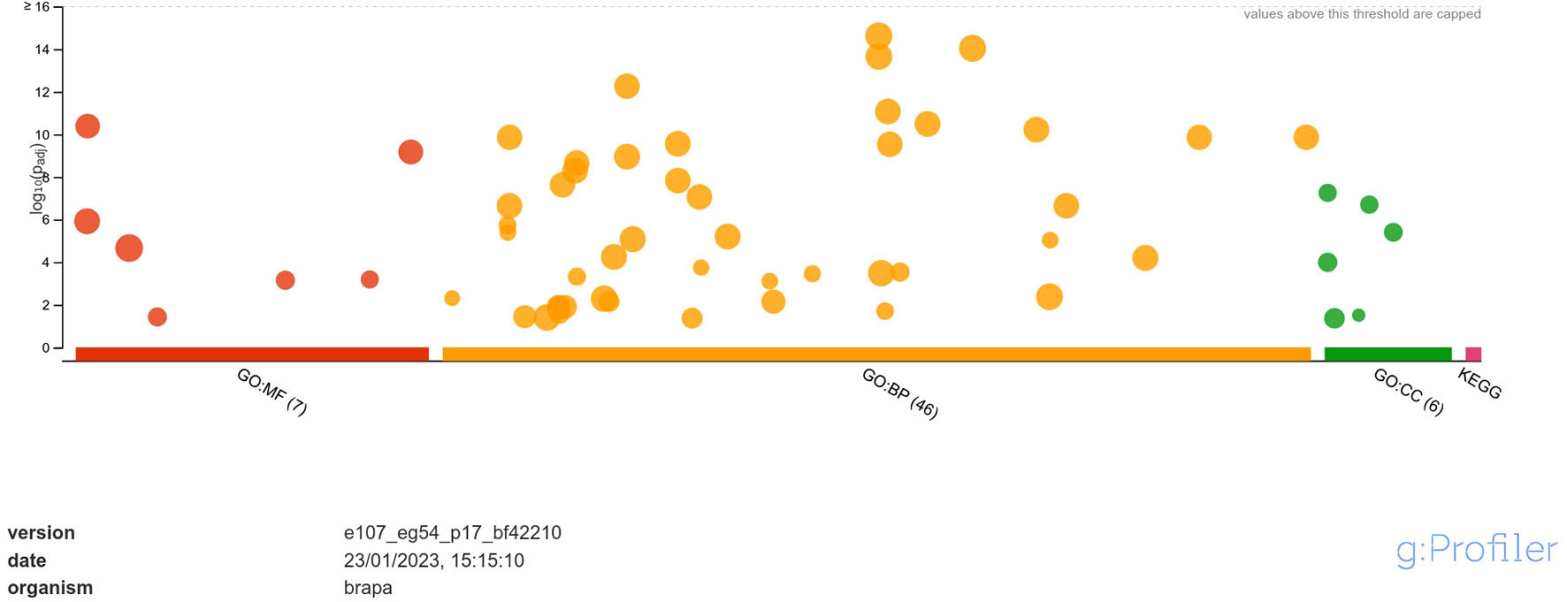
Functional enrichment analysis of DEGs downregulated in response to *A. brassicicola* infection, using g:Profiler. In the figure, the size of the circle represents the number of genes under the categories of MF, BP and CC.

The downregulated DEGs were annotated into 7 classes of MF namely, DNA-binding transcription factor activity, transcription regulator activity, DNA binding, protein binding, glutathione oxidoreductase activity, protein heterodimerization activity, disulfide oxidoreductase activity; 46 classes of BP namely, regulation of biological process, biological regulation, regulation of cellular process, regulation of nucleobase-containing compound metabolic process, regulation of nitrogen compound metabolic process, regulation of macromolecule metabolic process, regulation of primary metabolic process, regulation of RNA biosynthetic process, regulation of nucleic acid-templated transcription, regulation of DNA-templated transcription, regulation of cellular metabolic process, regulation of RNA metabolic process, regulation of metabolic process, regulation of macromolecule biosynthetic process, regulation of gene expression, regulation of cellular biosynthetic process, regulation of biosynthetic process, RNA biosynthetic process, nucleic acid-templated transcription, DNA-templated transcription, DNA-templated DNA replication, regulation of DNA replication, nucleobase-containing compound biosynthetic process, aromatic compound biosynthetic process, regulation of DNA-templated DNA replication, heterocycle biosynthetic process, organic cyclic compound biosynthetic process, regulation of DNA endoreduplication, regulation of cell cycle, response to stimulus, cell cycle DNA replication, regulation of cell cycle process, DNA endoreduplication, nucleic acid metabolic process, mitochondrial RNA metabolic process, RNA metabolic process, protein ubiquitination, response to chemical, response to endogenous stimulus, response to organic substance, response to hormone, regulation of DNA metabolic process, hormone-mediated signaling pathway, defense response, macromolecule biosynthetic process, protein modification by small protein conjugation and; 6 classes of CC namely, nucleosome, protein-DNA complex, DNA packaging complex, chromatin, extracellular matrix and chromosome (**Figure 4**).

### 3.4 Defense related genes upregulated in response to *A. brassicicola* infection

Upon inoculation with *A. brassicicola*, a large number of defense response genes were highly upregulated across 48 and 72 hpi in *S. alba* and *B. rapa*. The BLAST analysis of the DEGs revealed that numerous defense and resistance related genes particularly those related to signal transduction, receptor like protein kinases, ubiquitination, cell wall reinforcement, antioxidation, transcription factors (TFs), etc. were significantly upregulated in response to *A. brassicicola* infection. Some of the important genes include calmodulin, calcium binding protein, leucine-rich repeat transmembrane protein kinase, peroxidase, GSDL-motif lipase, WRKY, F-box, leucine-rich repeat, cytochrome related proteins, cyclin dependent protein kinase, ubiquitin protein ligase, glycosyl transferase, cellulose synthase, proline rich protein glycosyl hydrolase, expansin, auxin responsive protein, etc. Furthermore, 52 DEGs were found to be exclusively expressed in *S. alba* upon *A. brassicicola* infection. Some of the important defense related genes such as polygalacturonase inhibiting protein, ubiquitin-protein ligase, calcium-ion binding proteins/calmodulin-dependent protein kinase, cytochrome P450, peroxidase, ankyrin repeat family protein, etc. were exclusively expressed in *S. alba* across both the time points.

A large number of protein kinase genes were found to be upregulated commonly across both 48 and 72 hpi such as CDPK6, CDPK related kinase1, CRK1, SnRK2.4, SnRK, CIPK20, CYCP2, BSK3, BAM3, pfkB-type carbohydrate kinase family protein, LRR transmembrane protein kinase, etc. Out of the 35 protein kinase genes detected, CDPK6, glycerophosphodiester phosphodiesterase/kinase, pfkB-type carbohydrate kinase family protein and 2 protein kinase putative, were exclusively expressed in *S. alba*. In addition, we have identified 4 LRR protein kinase genes and 1 LRR protein that were highly induced during *A. brassicicola* infection across both the time points particularly at 48 hpi. Infection also resulted in the upregulation of several calcium sensing/calcium-ion binding proteins such as CAM7, CML38, CRK1, PSBQ-2, ATDEK1 etc. The expression of most of these genes was observed to be consistent across both the time points.

Several cell wall- related genes were also highly upregulated across the time period of study such as, xyloglucan endo-transglycosylase-related protein, expansin, endo-xyloglucan transferase, pectinase and polygalacturonase inhibitor proteins (PGIP) and pectin methylesterase inhibitors (PMEI). Among these, PGIP2 and XTR3 were exclusively upregulated in *S. alba*. Moreover, we have identified several CYP proteins such as, CYP71B3, CYP71B35, cytochrome B561-related, CYP71B14, CYP78A9 and CYP86A2. The genes CYP71B14 and CYP71B35 were exclusively expressed in *S. alba* across both the time points. Moreover, genes involved in lipid hydrolysis were also found to be highly upregulated in the present study. Several transcripts of lipases such as GDSL-motif lipase/hydrolase family protein, GLIP3; carboxylesterase/lipase, lipase class 3 family protein and family II extracellular lipase 1 were upregulated across the two time points upon infection by the necrotrophic pathogen. In addition, the upregulation of several ubiquitin-related genes was detected including ubiquitin-protein ligase-PUB22, PUB26, PB1, UPL4-ubiquitin-protein ligase, UBP11 and ubiquitin family protein. Out of these, UPL4 and UPB11 were exclusively expressed in *S. alba* in response to *A. brassicicola* infection.

A large number of TFs have also been found to be highly upregulated across the two time points after infection with *A. brassicicola.* We have identified a total of 24 TFs including ERF, WRKY, bZIP and MYB, which are known to play important roles in plant defense response against biotic and abiotic stresses. ERF1 was highly upregulated at 48 hpi whereas HB53 was found to be upregulated at 72 hpi. Besides, other classes of defense related genes such as auxin responsive gene, ankyrin repeat family protein, glycosyl hydrolase, 1-aminocyclopropane-1-carboxylate synthase (ACS8), MLP-like protein, glutaredoxin family protein etc. were also significantly upregulated upon infection.

### 3.5 Validation of differential gene expression

**Figure 5:**
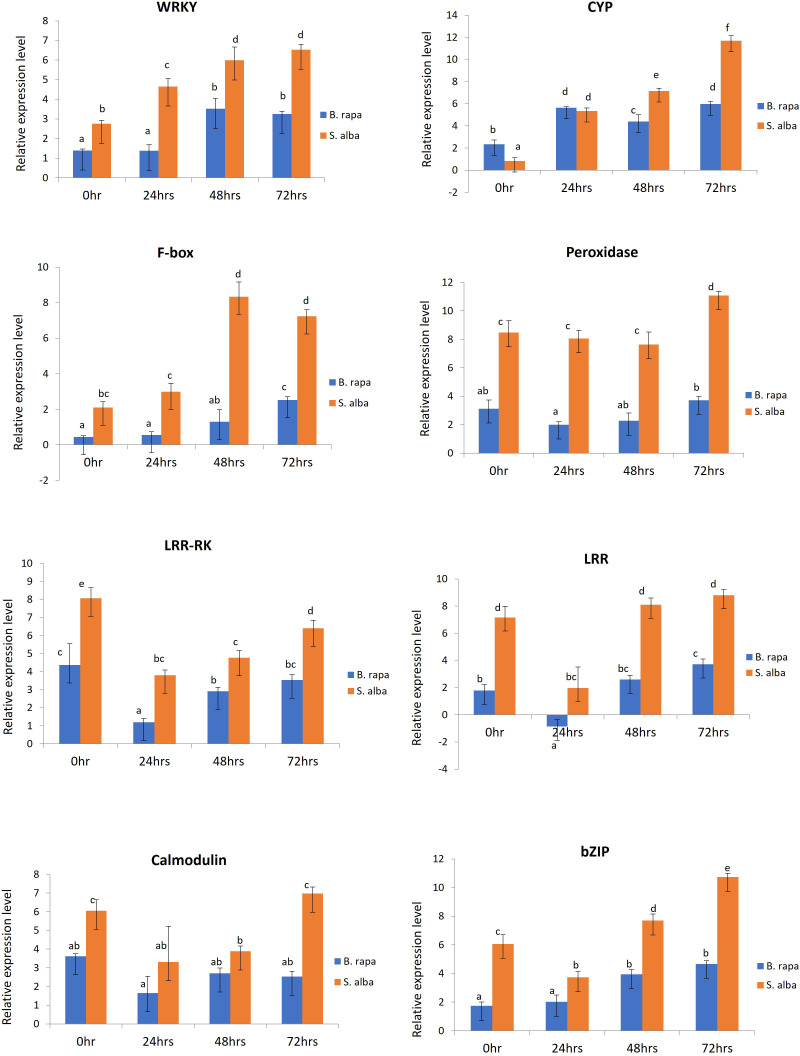
qPCR based expression profiling of selected genes in *B. rapa* and *S. alba*, induced upon artificial inoculation by *A. brassicicola*, across different time points (0, 24, 48 and, 72 hpi). Each reaction was performed thrice and the values represent the average of three technical replicates (analyzed by two-way ANOVA in Excel, p < 0.05).

In order to validate the RNA-seq based differential gene expression analysis, 8 defense related DEGs which were found to be significantly upregulated in *S. alba* upon *A. brassicicola* infection were chosen and their expression levels were profiled across the selected time points namely, 0, 24, 48 and 72 hpi, in both the resistant and susceptible germplasms by qPCR. The selected defense related genes were WRKY (WRKY domain protein), CYP (cytochrome P450), F-box (F-box domain protein), peroxidase, LRR-RK (leucine rich repeat transmembrane protein kinase), LRR (leucine-rich repeat), calmodulin, and bZIP (basic leucine zipper domain) (**Supplementary Data Sheet 3**). We were particularly interested in studying the differential expression patterns of these genes across the two contrasting germplasms. The qPCR study revealed that all the genes were activated upon infection by the pathogen and the expression patterns could be correlated with the RNA-seq data. We observed significantly higher transcript levels of majority of the genes in resistant *S. alba* compared to susceptible *B. rapa* (**Figure 5**). It was also observed that most of the defense response genes had a significantly higher basal expression level in *S. alba* as compared to *B. rapa*. Interestingly, the expression patterns of almost all the genes remained elevated in the resistant germplasm across all the time points, with significantly higher expression towards the later time point of 72 hpi.

Peroxidase (Bra023862) gene showed consistently higher expression pattern in *S. alba* as compared to *B. rapa* across the time course, starting from 0 hpi (8.47-fold), 24 hpi (8.052-fold) and 48 hpi (7.631-fold). Its expression further increased significantly at 72 hpi (11.067-fold) in *S. alba*. The expression of LRR-RK (Bra021758) and LRR (Bra004336) genes also showed a similar trend of significantly high expression in *S. alba* compared to *B. rapa* throughout the time points. The LRR-RK gene showed a very high expression in *S. alba* at 0 hpi (8.063-fold) that slightly lowered at 24 hpi (3.80-fold), but gradually increased towards 48 hpi (4.768-fold) and 72 hpi (6.40-fold). The LRR gene also showed a similar trend with significantly higher expression at 0 hpi (7.163-fold) which lowered at 24 hpi (1.972-fold), but again increased towards 48 hpi (8.102-fold) and 72 hpi (8.801-fold). Whereas in *B. rapa*, the expression of both these genes remained low all through. A very high expression of calcium sensor protein, calmodulin (Bra019453) gene was seen at 0 hpi (6.047-fold), that slightly lowered across 24 hpi (3.309-fold) and 48 hpi (3.883-fold) but again increased considerably at 72 hpi (6.972-fold) in *S. alba*.

The expression patterns of defense related TFs like bZIP (Bra007380) and WRKY (Bra000362) were also observed to be high post-inoculation. It was observed that throughout the time course, expression of WRKY increased gradually in *S. alba* starting from 2.753-fold at 0 hpi to 4.656-, 5.987- and 6.531-fold across 24 hpi, 48 hpi and 72 hpi respectively, which are significantly high compared to those in *B. rapa*. The expression of bZIP in *S. alba* remained high across all the time points with a fold change of 6.061 at 0 hpi, followed by 3.729-fold at 24 hpi. Its expression further increased by 7.691-fold at 48 hpi and 10.727-fold at 72 hpi. Comparatively, in *B. rapa*, bZIP was expressed at much lower levels.

The expression of two other important defense response genes cytochrome P450 (CYP) (Bra003019) and F-box (Bra031435) genes were also studied and validated by qPCR. In both the germplasms, the expression level of CYP increased at 24 hpi but significant induction could be seen at 48 hpi (7.147-fold) and 72 hpi (11.694-fold) in *S. alba* compared to *B. rapa*. In a similar manner, expression of F-box gene was significantly high in *S. alba* as compared to *B. rapa*. The gene showed high expression levels with fold changes of 2.102 and 2.987 across 0 hpi and 24 hpi respectively. It further increased towards 48 hpi (8.340-fold) and 72 hpi (7.237-fold) in *S. alba.*

## 4 Discussion

Plant-pathogen interactions consist of an interplay of complex interconnected biological pathways, and transcriptome profiling has facilitated the understanding of the molecular cues and cellular systems that are involved in these interactions (Wani and Ashraf, 2018; Neik et al., 2020). RNA-seq along with other advanced approaches has aided faster detection of candidate genes and dissection of intricate molecular processes implicated in plant defense against biotic stresses. For the first time, we report here an extensive RNA-seq based differential gene expression analysis among the resistant germplasm *S. alba* and the susceptible *B. rapa* during infection by the necrotrophic fungus *A. brassicicola*. The investigation detected a large number of defense or resistance related DEGs specifically modulated upon infection and studied their expression, particularly during the incompatible *S. alba* - A*. brassicicola* interaction in order to identify genes associated with resistance responses. Depending upon lesion development, from mid stage to distinct symptom development stage on *B. rapa*, we selected two time points i.e., 48 hpi and 72 hpi in order to identify the candidate genes involved in resistance against *A. brassicicola*. Eight such defense related genes were selected for validation of the transcriptomic data through qPCR across 0, 24, 48 and 72 hpi. A similar time period was selected to study the defense responses in *Brachypodium distachyon* upon infection by *Fusarium graminearum* and *Magnaporthe oryzae*, based on lesion development (Zhu et al., 2021a). In a study carried out by Chen et al. (2016) in two near isogenic lines of *B. rapa*, the clubroot susceptible ‘BJN3-2’ and resistant ‘CRBJN3-2’ in response to *P. brassicae*, 1875 genes were found to be upregulated and 2103 downregulated, across 0, 12, 72 and 96 hpi. Again in *B. oleracea*, gene expression analysis identified 885 DEGs between control and *F. oxysporum* f.sp. *conglutinans* infected roots of highly resistant R4P1, across 4, 12, 24 and 48 hpi (Xing et al., 2016). In *B. napus*, RNA-seq revealed that 584, 582, 526, 371, 822 and 1283 genes were upregulated at 1, 3, 6, 12, 24 and 48 hpi respectively, in response to *S. sclerotiorum* infection (Seifbarghi et al., 2017). In a similar study on *S. sclerotiorum* susceptible and resistant *B. napus* lines, 1301 and 1214 DEGs were found to be upregulated during 8-16 hpi and 24-48 hpi respectively in the susceptible line and, 1311 and 1335 DEGs were detected in the resistant line at the same time periods during interaction with the stem rot disease pathogen (Chittem et al., 2020). Compared to these plant-pathogen interaction studies on various *Brassica* spp., our investigation revealed a much higher number of transcripts that were specifically modulated upon inoculation. Thus, the study has been able to extensively dissect the defense transcriptomes of *A. brassicicola* infected resistant *S. alba* and susceptible *B. rapa*, and display the relative transcript abundances of various suites of defense related genes across the chosen time points.

### 4.1 Ca^2+^ signaling regulated defense responses

To defend against pathogens, plants have evolved a wide range of strategies of which Ca^2+^ signaling is a primary defense reaction. The rapid increase in Ca^2+^ levels in the cytoplasm in response to pathogen attack, along with Ca^2+^ sensors, play key roles in the activation of defense responses via expression of defense-related genes and hypersensitive response (HR). Some of the well identified plant defense signaling elements include CaM (calmodulin), CMLs (calmodulin-like proteins) and CaM-binding proteins (Ranty et al., 2006). Plant Ca^2+^ sensors have been classified into four main groups- Ca^2+^ dependent protein kinases (CDPKs), calcineurin B-like (CBL), the CaM group and the CML family (McCormack et al., 2005; Zhu et al., 2015). The CDPKs play an important role in plant immunity by mediating and transmitting defense signals in response to pathogen associated molecular proteins (PAMPs) and effector molecules (Liu et al., 2017). *Nicotiana tabacum* CDPK2 gene is reported to be activated when treated with fungal elicitor Avr9 in Cf-9 tobacco leaves (Romeis et al., 2000; Romeis et al., 2001). In the present study, several calcium sensing proteins such as CDPK6, CAM7, CML38, calcium ion binding, CaM binding and CDPK related kinase1, have been identified out of which CDPK6 was exclusively expressed in *S. alba* indicating its potential role in mediating resistance responses against *A. brassicicola*. The qPCR analysis showed that the calmodulin gene CAM7 (Bra019453) was highly upregulated upon infection in *S. alba* in comparison with *B. rapa*. These results indicate a rapid activation of calcium signaling in response to *A. brassicicola*, which might have greatly contributed towards initiating a strong defense response against the pathogen. In Arabidopsis and tobacco, the constitutive expression of soybean CMLs SCaM4 and SCaM5, led to the induction of pathogenesis-related (PR) genes and increased resistance to a vast range of pathogens (Ranty et al., 2006). According to another report, the overexpression of soybean SCaM-4/-5 in tobacco enhanced resistance to several pathogens including bacteria, fungi and viruses (Heo et al., 1999). Furthermore, in Arabidopsis, constitutive expression of SCaM-5 resulted in increased resistance to *Pseudomonas syringae* (Park et al., 2004). Besides, a study by Rao et al. (2014) states that overexpression of SCaM-4 in soybean enhances tolerance to two necrotrophic fungi namely, *Alternaria tenuissima* and *Phomopsis longicolla*, and *Phytophthora sojae* confirming that CaM/CMLs take part in plant immune responses. Recently, in rice, consistent upregulation of OsCBP60g-3, OsCBP60g-4, OsCBP60a and OsSARD-like1 genes was observed in response to *M. oryzae* as well as *Xanthomonas oryzae* (Kumari et al., 2022). The CaM-binding protein AtBAG6, has also been reported to induce programmed cell death in plants (Kang et al., 2006). This was seen in case of *S. alba* where necrosis was observed at the infection site without further progression of the disease lesion even after 7-10 dpi. Thus, an increase in the expression of calcium signaling and calmodulin genes might have triggered the expression of defense related genes leading to HR in *S. alba* against *A. brassicicola*.

### 4.2 Leucine rich repeat-receptor like kinase mediated defense response

The leucine rich repeat-receptor like kinases (LRR-RKs) constitute the largest family of receptor proteins in plants. Various reports have suggested that in addition to LRR-RKs, RLPs are essential for plant innate immune response and development (Stergiopoulos and de Wit, 2009; Belkhadir et al., 2014). These kinases play an active role in recognizing PAMPs thereby regulating resistance responses to fungal invasion. In our study, the expressions of LRR-RK (Bra021758) as well as the recognition domain LRR (Bra004336) were found to be highly induced in *S. alba* in comparison with *B. rapa*. A similar induction of an uncharacterized LRR-RLK gene (7-fold) was reported in case of “uzu” barley in response to infection with *F. culmorum* at 48 hours post-fungal treatment (Ali et al., 2014). In another study, gene expression studies revealed that, wheat TaLRRK-6D was highly expressed in response to *F. graminearum* invasion and its mycotoxic virulence factor deoxynivalenol (Thapa et al., 2018). Again, in wheat, the gene TaRLP1.1 was reported to be involved in resistance against stripe rust caused by *Puccinia striiformis* f.sp. tritici (Jiang et al., 2013b). In rice, the LRR-RLP gene OsRLP1, was found to be significantly induced upon infection with rice black-streaked dwarf virus (Zhang et al., 2021a). Moreover, transcriptome studies have demonstrated the involvement of RLKs, RLPs along with WAKLs and TIR-NBS in *B. napus*-*Leptosphaeria maculans* interaction (Becker et al., 2017).

In our study, the expression of LRR gene was significantly upregulated in resistant *S. alba* immediately after infection. However, as the plant tried to cope up with pathogen invasion, the expression of the gene lowered at 24 hpi but later increased again after 48 hpi. Upregulation of LRR genes was observed in resistant chickpea cultivar, CDC Luna and CDC Corinne at 24, 48 and 72 hpi after infection with *Ascochyta rabiei* (Sagi et al., 2017). The LRR regions in resistance genes activate signal transduction in plants thereby inducing the expression of defense-related genes (Shanmugam, 2005). In tomato, the induction of NBS-LRR gene was reported to be positively correlated with resistance against *Phytophthora infestans* (Jiang et al., 2018). Moreover, the CC-NBS-LRR gene RppM, has been found to confer resistance to southern corn rust (Xu et al., 2018). Similarly in rice, the NBS-LRR protein PID3, interacts with OsRac1 to activate the expression of transcription activator RAI1, resulting in resistance against *M. oryzae* (Zhou et al., 2019). In a recent study, soybean NBS-LRR gene Rps11 was reported to be involved in broad spectrum resistance to *P. sojae* (Wang et al., 2021). Earlier studies had also reported that upregulation of NBS-LRR genes activate defense responses in *S. alba* and *Brassica* spp. upon infection by Alternaria pathogens (Ghose et al., 2008; Fatima et al., 2019b). Thus, higher transcript levels of LRR along with LRR-RK in *S. alba* across all the time points in our study, clearly indicate their involvement in resistance against *Alternaria*.

### 4.3 Signaling pathways mediated by protein kinases

In addition to CDPKs and LRR-RKs, several other classes of protein kinase genes have been observed to be significantly upregulated during the *A. brassicicola*- *S. alba/ B. rapa* interaction. This demonstrates their roles in mediating crucial signaling pathways for defense against the necrotroph. Protein kinases catalyze reversible phosphorylation and modulate the key processes required for activation of plant defense responses through coordinating signaling networks during pathogen recognition (Romeis et al., 2001; Jiang et al., 2022). The cysteine rich receptor-like kinases (CRKs) are a major class of plant receptor like kinases that have been reported to perform key roles in cell death and disease resistance (Quezada et al., 2019). In our study, a CRK gene was noticeably upregulated across both the time points. In addition, previous studies had identified two pfkB type carbohydrate kinases, FLN1 and FLN2 in *S. alba* and *A. thaliana* as the components of the thylakoid bound PEP complex (Gilkerson et al., 2012). The exclusive expression of a pfkB type carbohydrate kinase was also observed in *S. alba* in our study that suggests its potential role in mediating defense responses. The sucrose non-fermentation-related protein kinase, SnRK, is a Se/Thr protein kinase that plays a major role in plant stress response by phosphorylating target proteins during signaling pathways (Wang et al., 2019). Moreover, the CIPKs are Ser/Thr protein kinases, classified to SNF1-related kinases 3 (SnRK3), which are targeted by CBL to take part in calcium signaling (Ma et al., 2020). The significant upregulation of several Ser/Thr protein kinase genes in the study depicts that these enzymes have a crucial role to play in activating defense responses against *A. brassicicola*. Additionally, we have noticed high upregulation of a glycerophosphodiester phosphodiesterase kinase (GDPD) gene. Its exclusive expression in *S. alba* indicates that it might have played a major role in conferring resistance to *A. brassicicola*.

### 4.4 Cell wall modification

The plant cell wall acts as a physical barrier, the first line of defense, to invasion by microbial pathogens where a number of changes occur in response to pathogen attack (Malinovsky et al., 2014). The necrotrophic fungi synthesize diverse enzymes to degrade the cell wall matrix. In order to combat this, plant cell wall maintains its integrity by inhibiting cell wall damaging enzymes via expressing several enzymes such as PGIP (polygalacturonase inhibiting protein) (Khandagale et al., 2022). The PGIPs protect the cell wall from pathogen attack with the help of polygalacturonases (Kalunke et al., 2015). PGIPs also protect pectin from degradation and lead to the synthesis of compounds that can be recognized by damage associated molecular patterns (DAMPs) which ultimately activate PTI, thereby slowing down pathogen colonization (Federici et al., 2006). PGIP2 was found to be exclusively and noticeably expressed in the resistant germplasm *S. alba* in response to *A. brassicicola* infection in our study, which suggests its potential role in preventing pathogen colonization. Upregulation of such genes have also been observed in response to *Alternaria porri* in onion (Khandagale et al., 2022). In addition, several genes encoding xyloglucan endotransglycosylase/hydrolase (XTH) have been upregulated in the study. The plant XTHs are involved in cell wall remodeling and expansion, and several XTH genes have been reported to be differentially expressed in response to fungal infection in plants (Sharmin et al., 2012; Stratilova et al., 2020; Niraula et al., 2021). Additionally, genes for other important cell wall associated proteins such as hydroxyproline-rich glycoprotein, proline-rich protein and glycine-rich protein were also highly upregulated in the present study. Such proteins are important structural components of the cell wall with potential antimicrobial properties (Deepak et al., 2010; Halder et al., 2019). The cross-linking of hydroxyproline-rich glycoproteins, in particular, strengthens the cell walls thus preventing pathogen colonization thereby, significantly contributing to defense against infection (Deepak et al., 2007; Deepak et al., 2010). Thus, these genes are likely to play key roles in conferring resistance responses against *A. brassicicola* through cell wall modification and reinforcement.

### 4.5 Cytochrome P450s as important regulators of defense

In plants, the cytochrome P450s (CYPs) constitute the largest family of enzymes that are implicated in diverse biological and biosynthetic processes, primarily, the detoxification of xenobiotics and plant defense responses to stresses. These enzymes are involved in the biosynthesis of antioxidants, pigments, signaling molecules, cell wall components, fatty acids and, defense related compounds or secondary metabolites such as alkaloids, flavonoids, phenylpropanoids, phytoalexins, etc. (Pandian et al., 2020). In our study, several CYPs including CYP71B3, CYP71B35, CYP71B14, CYP78A9, CYP86A2, were detected to be significantly upregulated in response to *A. brassicicola* infection during the course of study, especially at the later stage of infection. Moreover, the qPCR analysis revealed that CYP71B14 (Bra003019) was upregulated by 7.147-fold at 48 hpi and further by 11.694-fold at 72 hpi, in *S. alba*. These results indicate that CYPs have a significant role to play in generating a strong defense response against *A. brassicicola*. The CYP proteins are involved in the biosynthesis of cell wall components and epicuticular wax which act as the primary structural barrier against pathogen attack in plants (Pandian et al., 2020). Moreover, synthesis of two phytoalexins- sinalbins A and B has already been reported to be produced in *S. alba* against *A. brassicae*, that are associated with partial resistance (Pedras and Zaharia, 2000). Several reports state that the CYP450 genes play a crucial role in jasmonic acid (JA) induced immunity and are involved in disease resistance against necrotrophic as well as other pathogens. In Arabidopsis, the CYP450 protein CYP82C2 regulates JA-induced resistance to necrotrophic fungus *Botrytis cinerea* (Liu et al., 2010). Similarly, the cotton GhCYP82D gene reportedly imparts resistance to pathogens by modulating the octadecanoid pathway (Sun et al., 2014). A recent report states that rice CYP protein CYP716A16 is involved in broad spectrum resistance to the necrotroph *Rhizoctonia solani* and hemibiotroph *X. oryzae* pv. *oryzae*, through regulation of JA-dependent defense signaling and ROS levels (Wang et al., 2022). Hence, high expression of CYP genes demonstrates their active role in resistance against the necrotrophic fungus *A. brassicicola*, most likely through a JA-dependent pathway by way of production of antimicrobials and reinforcement of cell wall.

### 4.6 Regulation of ROS and antioxidant defense

A class of PR proteins consists of peroxidases, which act as antioxidant enzymes associated with oxidative stress responses during initial infection process. These enzymes are involved in cross-linking of call wall polymers or initiation of signaling pathways ultimately leading to HR and PR gene expression (Mir et al., 2015; Kaur et al., 2022). In the present study, we have found the exclusive expression of peroxidase 30 gene (Bra023862) upon inoculation by the necrotrophic pathogen. Moreover, in the qPCR analysis, we detected a very high level of expression of the peroxidase gene in *S. alba* as compared to *B. rapa*. These results clearly indicate its defense related function against infection by the Alternaria pathogen. A class III peroxidase family gene, AtPRX53, was shown to be upregulated during *Heterodera schachti* infection in Arabidopsis (Puthoff et al., 2003; Szakasits et al., 2009). Similarly, upregulation of peroxidase genes was observed in rice upon infection with *M. oryzae* (Mir et al., 2015). In apple, upregulation of peroxidase genes was reported in response to *A. alternata* causing Alternaria blotch disease (Zhang et al., 2015). Additionally, other related enzymes such as glutathione s-transferase, glutaredoxin and catalase have also been upregulated in our study which suggests their crucial role in defense against the necrotroph.

### 4.7 Lipases associated with defense

Lipids maintain structural integrity of cells and act as signal transduction mediators at the host-pathogen interface thus, play important roles in host-pathogen interactions especially in the induction of systemic acquired resistance (Gao et al., 2015; Mehta et al., 2021). Plant immunity associated with lipids involves the activation of lipases, the lipid hydrolyzing enzymes, that breakdown or convert lipids into subcellular compartments (Lee and Park, 2019). The GDSL lipases (GLIPs) are a subclass of lipolytic enzymes that are characterized by a GDSL motif and are reported to play major roles in plant immunity, mainly in rice and Arabidopsis (Oh et al., 2005; Lee et al., 2009; Gao et al., 2017). AtGLIP1, the GLIP of Arabidopsis, has been found to possess antimicrobial activity and regulate resistance to *A. brassicicola* in combination with ethylene signaling (Oh et al., 2005; Kwon et al., 2009). Similarly, both GLIP1 and GLIP3 have been shown to provide resistance to *Botrytis cinerea* in Arabidopsis (Han et al., 2019). Our results also indicate significant upregulation of lipase genes such as GDSL-motif lipase/hydrolase family protein and GLIP3, which clearly suggests their possible involvement in generating defense responses against *A. brassicicola*.

### 4.8 TFs in defense transcriptional reprogramming

Several TFs are key regulators of plant immune system and are involved in the regulation of PTI and ETI. The common families of TFs implicated in plant resistance responses against pathogens include WRKY, bZIP, NAC, AP2/ERF (Apetala2/Ethylene Responsive Factor) and bHLH (basic helix-loop-helix) (Pandey and Somssich, 2009; Campos et al., 2022). Pathogens invading the plant cell wall trigger the activation of phenylpropanoid pathway for plant defense. Phenylpropanoids are antimicrobial compounds that are induced, and play crucial roles during plant−pathogen interactions (Naoumkina et al., 2010). The phenylpropanoid pathway is regulated by TFs such as WRKY, MYB, bZIP, NAC etc. (Meng et al., 2021b). In the current study also, we have observed upregulation of WRKY and MYB across both the time points. The qPCR analysis further revealed significantly high expression of one WRKY family transcription factor (Bra000362) in *S. alba* as compared to *B. rapa*. The Arabidopsis AtWRKY22 and AtWRKY29 have been reported to be important components of MAPK (mitogen activated protein kinase) regulated defense responses against pathogens (Asai et al., 2002). The upregulation of several WRKY genes was also observed in seedlings of *B. distachyon* inoculated with *F. graminearum* and *M. grisea* (Wen et al., 2014). Likewise in rice, overexpression of WRKY22 gene enhanced resistance to *Pyricularia oryzae* indicating its role as a positive regulator of defense (Cheng and Wang, 2014). Recently, Shen et al. (2023) have reported the upregulation of WRKY33 in *B. oleracea* during *A. brassicicola* infection in broccoli lines. WRKY33 reportedly confers resistance to necrotrophic pathogens and regulates the indolic glucosinolate metabolic pathway leading to resistance against *A. brassicicola* in Arabidopsis and *Brassica* crops (Zheng et al., 2006; Tao et al., 2022). In a similar manner, two bZIP TFs namely, ATBZIP2 and BZIP61 were upregulated in our study, in response to *Alternaria* infection. One bZIP gene (Bra007380) was found to exhibit significantly higher expression in the qPCR analysis, which demonstrates its crucial role in defense against the necrotrophic pathogen. The bZIP TFs are reported to play active roles in induction of resistance in Arabidopsis (Jakoby et al., 2002; Lee et al., 2006). Overexpression of MebZIP3 and MebZIP5 has been found to enhance callose deposition leading to improved resistance to cassava blight (Li et al., 2017). Enhanced resistance to *S. sclerotiorum* and *P. sojae* has been observed in transgenic soybean through overexpression of the GmbZIP15 gene (Zhang et al., 2021b). StbZIP61 and StNPR3L have been reported to regulate salicylic acid-mediated resistance against *P. infestans* infection in potato (Zhou et al., 2018). In addition, we observed significant upregulation of two MYB and one ERF gene upon *A. brassicicola* infection. These TFs also play important roles in regulating stress responses in plants, principally as activators of PR gene expression (Al-Attala et al., 2014; Zang et al., 2020). The overexpression of Arabidopsis AtERF96 has been reported to enhance resistance to necrotrophic pathogens such as *Botrytis cinerea* and *Pectobacterium carotovorum* (Catinot et al., 2015). Similarly in maize, overexpression of ZmERF105 reportedly improved resistance to *Exserohilum turcicum* and the lines were found to show enhanced PR gene expression along with higher activity of superoxide dismutase and peroxidase (Zang et al., 2020). In case of wheat, TaMYB29 has been reported to activate defense against the stripe rust fungus through H_2_O_2_ accumulation and PR gene expression (Zhu et al., 2021b). Remarkably, several TFs and some downstream phenylpropanoid pathway genes have been observed to be commonly downregulated in *S. alba* and *B. rapa* during the period of the current study. Further research particularly at later time points after inoculation would only establish their expression trend with progress of infection. Zheng et al. (2019) studied the basal and cultivar specific resistance of *B. napus* towards *V. longisporum* and detected an overall increase in phenylpropanoid synthesis and expression of key genes of the pathway starting at 7 dpi (days post inoculation), which is quite late compared to the time points studied in the present investigation. It is therefore likely that the TFs and the genes implicated in phenylpropanoid biosynthesis could be upregulated at the later stages of infection by *A. brassicicola*. Moreover, a number of WRKY, NAC and MYB TFs have also been reported to act as negative regulators of plant defense, particularly the phenylpropanoid pathway, and are targeted by pathogens to enhance plant susceptibility (Liu et al., 2015; Seo et al., 2015; Yuan et al., 2019; Cao et al., 2020).

## 5 Conclusion

The study has generated a significant amount of information about the genes regulated during the interactions between the necrotroph *A. brassicicola* and the resistant cultivar *S. alba*, as well as susceptible *B. rapa*. It has also resulted in the identification of numerous gene candidates related to defense thereby providing insights into mechanisms underlying the interaction with *A. brassicicola*. Furthermore, the analysis demonstrated that the differences between resistance and susceptibility against infection by the necrotroph could be associated with a host-specific regulation of expression of a suite of genes involved in pathogen recognition, signal transduction, cell wall modification, antioxidation, transcription regulation and biosynthesis of defense-related proteins. Complete functional characterization of the key defense response genes could be done in the future, as they would make excellent candidates for generating Alternaria-resistant Brassica varieties.

## Data availability statement

The datasets presented in this study can be found in online repositories. The names of the repository/repositories and accession number(s) can be found below: Bioproject ID: PRJNA784760.

## Author contributions

RA performed the experiments, interpreted the results and wrote the manuscript; KKD and MKM analyzed the data; MS-K, MKM and BKS provided intellectual inputs to the study and reviewed the manuscript; PB conceived, designed and supervised the study, revised and edited the manuscript. All authors contributed to the article and approved the submitted version.
