# Supplementary material for: Comparative transcriptome profiling reveals differential defense responses among Alternaria brassicicola resistant Sinapis alba and susceptible Brassica rapa
Source: Front Plant Sci. 2024 Jan 18;14:1251349. doi: 10.3389/fpls.2023.1251349 (PMC10831657; doi:10.3389/fpls.2023.1251349)
Supplement: Supplementary file 4 [file Table_1.docx]

Supplementary table 1: Genes selected for qPCR analysis along with the details of primers

| Gene ID | Gene code (name) | Forward primer  (5'-3') | Reverse primer  (5'-3') | Annealing temperature  (°C) |
| --- | --- | --- | --- | --- |
| Bra000362 | WRKY (WRKY domain protein) | TCTTCTCTCTCCTCTCCTTCTT | GTCCTCCACCCTGAAGATTATT | 60 |
| Bra003019 | CYP (Cytochrome P450) | TGGTAGTGGAAGGAGGATGT | GCTTCCAATCGAAACGGTAAAG | 60 |
| Bra031435 | FBOX (F-box domain protein) | GGTGTGGAAGAAGGAGGATTAG | ACCGAATCTCTTGTCTCTGAAAT | 60 |
| Bra023862 | Peroxidase | CAGAGGATTCGGTTTCGTAGAG | GAGCGGTCAAAGCGATGATA | 60 |
| Bra021758 | LRR-RK (Leucine rich repeat-protein kinase) | AAAGTCCGGTTCCGGTTATG | GTTGTTGGGTTTCTTCGTTGAG | 60 |
| Bra004336 | LRR (Leucine rich repeat) | TTCATCACTAGCCTCACCAATC | CGGTTTCCAGACAGCACTAA | 60 |
| Bra019453 | Calmodulin | TCAGCTTCAGTTGGGTTCTG | CTATTCGACAAGGACGGAGATG | 60 |
| Bra007380 | bZIP (Basic leucine zipper domain) | GAACAACCACCGTCCGATAATA | GCATTGGCTTTGAACCTCATC | 60 |
| ACT | ACT2 (Actin 2) | TGGGTTTGCTGGTGACGAT | TGCCTAGGACGACCAACAATACT | 60 |
